# Analysis of High-Risk Factors and Construction of a Nomogram Predictive Model for Deep Venous Thrombosis in Pelvic and Acetabular Fracture Patients Treated Conservatively

**DOI:** 10.7759/cureus.56091

**Published:** 2024-03-13

**Authors:** Xiaobo Fan, Zongyou Yang, Yuan Liu, Zhikun Wei, Chenyang Zhao, Chaojian Pang, Zhihong Wang, Hongcheng Yang

**Affiliations:** 1 Department of Orthopedic Surgery, The First Hospital of Handan, Handan, CHN; 2 Department of Orthopedic Surgery, The Third Hospital of Hebei Medical University, Shijiazhuang, CHN

**Keywords:** nomogram model, risk factors‎, conservative treatment, acetabula, pelvis, thrombosis

## Abstract

Purpose: This study aims to develop a predictive nomogram model to assist physicians in making evidence-based decisions and potentially reduce the incidence of deep venous thrombosis (DVT).

Methods: We conducted a retrospective study, including patients admitted to the hospital from January 2014 to January 2022 with a closed, single pelvic or acetabular fracture. Comprehensive data were collected for each patient, encompassing demographics, injury characteristics, comorbidities, and results from laboratory tests and lower extremity ultrasounds. Potential risk factors were identified by univariate and multivariate logistic regression analyses. The predictive model was constructed and then internally validated. Calibration accuracy was assessed using a calibration slope and the Hosmer-Lemeshow goodness-of-fit test. The discrimination of the nomogram model was evaluated using the C-statistic.

Results: Out of 232 individuals who underwent conservative treatment, 57 (24.6%) were classified into the DVT group and 175 (75.4%) into the non-DVT group based on lower extremity ultrasound findings. Predominantly, patients were aged between 41 and 65 in both groups. Body mass index (BMI) comparison showed that 54.29% (95/175) of the non-DVT group fell within the healthy weight range, while 45.61% (26/57) in the DVT group were overweight. Notably, the proportion of obesity in the DVT group was more than double that in the non-DVT group, indicating a higher DVT risk with increasing BMI (P=0.0215). Lower red blood cell (RBC) counts were observed in DVT patients compared to non-DVT ones (P<0.001). A similar pattern emerged for D-dimer, a marker for blood clot formation and dissolution, with significant differences noted (P=0.029). Multivariable analysis identified age, BMI, associated organ injury (AOI), American Society of Anesthesiologists score, hemoglobin (HGB), RBC, and D-dimer as candidate predictors. Significant variables included age (OR, 3.04; 95% CI, 1.76-5.26; P<0.001), BMI (OR, 1.97; 95% CI, 1.22-3.18; P=0.006), AOI (OR, 2.05; 95% CI, 1.07-3.95; P=0.031), and HGB (HR, 0.59; 95% CI, 0.39-0.88; P=0.010). The discrimination was 0.787, with a corrected c-index of 0.753. Calibration plots and the Hosmer-Lemeshow test indicated a good fit (P=0.7729). Decision curve analysis revealed a superior net clinical benefit when the predicted probability threshold ranged from 0.05 to 0.95.

Conclusions: We developed a nomogram predictive model, and it could act as a practical tool in clinical workflows to assist physicians in making favorable medical decisions, which potentially reduces the incidence of DVT in those patients with pelvic and acetabular fractures treated conservatively.

## Introduction

Deep venous thrombosis (DVT) is a common and severe complication in patients with traumatic fractures. It is reported that over one-third of patients with pelvic and acetabular fractures will develop DVT postoperatively [1,2]. Early detection and prediction of DVT are crucial for medical decisions and reducing its incidence. Several studies have focused on predicting DVT in patients undergoing surgical procedures [3-5]. Patients receiving nonoperative treatments would be more likely to experience longer periods of lower limb immobilization, which was associated with a higher risk of thrombotic complications. In addition, individuals receiving conservative treatment for pelvic and acetabular fractures may face challenges accessing consistent, standardized monitoring and testing at home. The absence of regular evaluation and diagnostic procedures increases the likelihood of developing pulmonary embolism (PE) as a consequence of DVT. Therefore, we conducted a retrospective cohort study to identify the factors relevant to the development of DVT. Furthermore, we plan to develop a nomogram model to predict DVT.

## Materials and methods

We included a cohort of adult patients with a closed and single pelvic and acetabular fracture who were admitted to the hospital from January 2014 through January 2022. The design and protocol of the research were approved by the Ethics Committee of the Third Hospital of Hebei Medical University (approval number: 2019-18-01). This study was conducted in accordance with the ethical guidelines and regulations of the Research Ethics Committee. Patient confidentiality and privacy were strictly maintained during data collection and analysis.

Data collection was conducted by reviewing the electronic medical record system and the picture archiving/communication system. We excluded patients with pathological fractures, unstable vital signs, incomplete data, or those managed surgically from further analysis. Comprehensive information was extracted for each patient, including demographic data such as age, sex, tobacco consumption, height, and body weight. Based on age, subjects were categorized into three groups: young (18-40 years), middle-aged (41-65 years), and old (>65 years). The body mass index (BMI) was calculated using height and body weight, and patients were further classified into four categories: underweight (<18.5), healthy weight (18.5-24.9), overweight (25-29.9), and obese (BMI ≥30). Injury characteristics involved the American Society of Anesthesiologists (ASA) classification for assessing injury severity, mechanism of injury, and time from injury to admission. Patients arriving at the institution more than 24 hours post-injury were considered to have delayed admission. Associated organ injuries (AOI) included the craniocerebrum, lung, spleen, intestines, or urinary system, with patients having multiple organ injuries classified into a multi-organ injury group. It was noted whether patients were administered anticoagulant drugs such as low-molecular-weight heparin or rivaroxaban. Upon admission, a series of laboratory tests was immediately performed, and all individuals received routine venous duplex ultrasonography surveillance for DVT. The laboratory markers that were analyzed included red blood cell (RBC), white blood cell (WBC), hemoglobin (HGB), triglyceride (TG), platelet (PLT), prothrombin time (PT), activated partial thromboplastin time (APTT), international normalized ratio (INR), fibrinogen (FIB), and D-dimer levels. Based on the World Health Organization classification, we assessed the severity of anemia upon admission and subclassified patients into non-anemia (HGB >120 g/L for females, >130 g/L for males), mild anemia (HGB 110-119 g/L in women and 110-129 g/L in men), moderate anemia (HGB <110 g/L), or severe anemia (HGB <80 g/L) [6].

Statistical analysis

We analyzed the collected data to construct a predictive model for DVT in pelvic and acetabular fracture patients undergoing conservative treatment. The mean and standard deviation were used to summarize continuous demographic and clinical characteristics, while percentages were used for categorical variables. The association between each factor and DVT was examined using two-sample t-tests or Mann-Whitney U tests. Predictors with p-values less than 0.10 were included in the multivariable logistic regression analysis, which employed a backward stepwise elimination method. This process led to the construction of the predictive model. The discrimination of the model was measured by the C-statistic. Calibration accuracy was assessed using a calibration slope with 1,000 bootstrap iterations and tested via the Hosmer-Lemeshow goodness-of-fit test. Additionally, decision curve analysis (DCA) was applied to evaluate the clinical value of the nomogram predictive model. All analyses were conducted using SPSS Statistics version 25.1 (IBM Corp. Released 2017. IBM SPSS Statistics for Windows, Version 25.1. Armonk, NY: IBM Corp.) and R version 4.2.0 (The R Foundation, IN, USA).

## Results

Clinical characteristics

The retrospective cohort study included a total of 232 patients based on the screening criteria who were treated conservatively at the Third Hospital of Hebei Medical University between January 2014 and January 2022. The demographic characteristics are listed in Table 1. The age range was between 18 and 91 years, with a mean age of 52.47 years (SD, 16.710). BMI ranged from 17.6 to 37.5 kg/m², with an average BMI of 24.499 (SD, 3.163). Car crashes were the most common mechanism of pelvic and acetabular fractures. Out of the 232 individuals who underwent conservative treatment, 57 (24.6%) were diagnosed with DVT. The comparison of the two groups is also presented in Table 1.

**Table 1:** Characteristics of patients with pelvic and acetabular fractures treated conservatively. Values are presented as numbers (%) AOI: associated organ injury, ASA: American Society of Anesthesiologists, DVT: deep venous thrombosis, BMI: body mass index

Factor	non-DVT (n=175)	DVT (n=57)	P-value
Age (yr)			<0.001
18-40	56 (32%)	4 (7.02%)	
41-65	89 (50.86%)	29 (50.88%)	
>65	30 (17.14%)	24 (42.11%)	
Sex			0.4381
Female	62 (35.43%)	17 (29.82%)	
Male	113 (64.57%)	40 (70.18%)	
BMI			0.0215
Underweight	2 (1.14%)	1 (1.75%)	
Normal weight	95 (54.29%)	18 (31.58%)	
Overweight	59 (33.71%)	26 (45.61%)	
Obesity	19 (10.86%)	12 (21.05%)	
Tobacco consumption			0.3241
No	148 (84.57%)	45 (78.95%)	
Yes	27 (15.43%)	12 (21.05%)	
Coagulation disorders			0.3518
No	168 (96%)	53 (92.98%)	
Yes	7 (4%)	4 (7.02%)	
Mechanism			0.4738
Car crash	74 (42.29%)	25 (43.86%)	
Fall from height	25 (14.29%)	12 (21.05%)	
Ground-level fall	57 (32.57%)	12 (21.05%)	
Struck by objects	20 (11.43%)	7 (12.28%)	
AOI			<0.001
No organ injury	138 (78.86%)	34 (59.65%)	
Single-organ injury	35 (20%)	15 (26.32%)	
Multi-organ injury	2 (1.14%)	8 (14.04%)	
Delayed admission			0.1779
No	138 (78.86%)	40 (70.18%)	
Yes	37 (21.14%)	17 (29.82%)	
Use of anticoagulant			0.1223
No	34 (19.43%)	53 (92.98%)	
Yes	141 (80.57%)	4 (7.02%)	
ASA score			0.0241
1	30 (17.14%)	4 (7.02%)	
2	109 (62.29%)	32 (56.14%)	
3	34 (19.43%)	21 (36.84%)	
4	2 (1.14%)	0 (0%)	

Significant differences were observed in age, BMI, AOI, and ASA score. Although the majority were within the 41-65 age group in both cohorts, a notable inclination towards the >65 age group was discerned in the DVT cohort. The comparison of BMI revealed that the predominant number (54.29%, 95/175) of the non-DVT cohort was within the normative weight range, whereas it was within the overweight range (45.61%, 26/57) in the DVT cohort. The proportion of obesity in the DVT group was more than twice that in the non-DVT group, suggesting increased susceptibility to DVT with a higher BMI (P=0.0215). Of the patients without DVT, there was one patient associated with a craniocerebrum injury, 20 with a lung injury, two with a spleen injury, one with an intestine injury, 11 with a urinary system injury, and two with a multi-organ injury. There was one with a craniocerebrum injury, 13 with a lung injury, one with a urinary system injury, and eight with a multi-organ injury. The ASA score also appeared to have a marked impact on DVT risk, with the score 3 group exhibiting a higher rate in the DVT cohort and the score 1 group exhibiting a lower rate compared to their counterparts in the non-DVT cohort (P=0.0241). Blood test findings are presented in Table 2.

**Table 2:** Blood test results of patients with pelvic and acetabular fractures were treated conservatively. Values are presented as mean ± standard deviation or number (%) DVT: deep venous thrombosis, HGB: hemoglobin, RBC: red blood cell, WBC: white blood cell, TG: triglyceride, PLT: platelet, PT: prothrombin time, APTT: activated partial thromboplastin time, INR: international normalized ratio, FIB: fibrinogen

Factor	non-DVT (n=175)	DVT (n=57)	P-value
HGB			<0.001
Non-anemia	66 (37.71%)	7 (12.28%)	
Mild	47 (26.86%)	13 (22.81%)	
Moderate	55 (31.43%)	27 (47.37%)	
Severe	7 (4.00%)	10 (17.54%)	
RBC (10^12^/L)	3.91+0.64	3.54+0.58	<0.001
WBC (10^9^/L)	3.54+0.58	9.52+3.72	0.661
TG (mmol/L)	9.27+3.24	1.12+0.44	0.865
PLT (10^9^/L)	9.52+3.72	184.75+88.74	0.520
PT (s)	1.13+0.58	12.24+1.2	0.469
APTT (s)	1.12+0.44	28.94+4.07	0.192
INR	192.94+62.63	1.09+0.09	0.921
FIB (g/L)	184.75+88.74	3.4+1.45	0.477
D-dimer (mg/L)	12.38+1.28	8.27+12.56	0.029

HGB, RBC, and D-dimer played a significant role in the prevalence of DVT among patients. In terms of HGB, the majority of the non-DVT cohort (37.71%, 66/175) was ensconced within the non-anemia patients, whereas the DVT cohort displayed a higher proportion in the moderate anemia group (47.37%, 27/57). The RBC in patients detected with DVT was notably lower than in those without DVT (P<0.001). The same trend was observed with D-dimer levels, indicators of blood clot formation and dissolution in the body commonly used to assess thrombus formation and dissolution (P=0.029).

Model development

**Figure 1:**
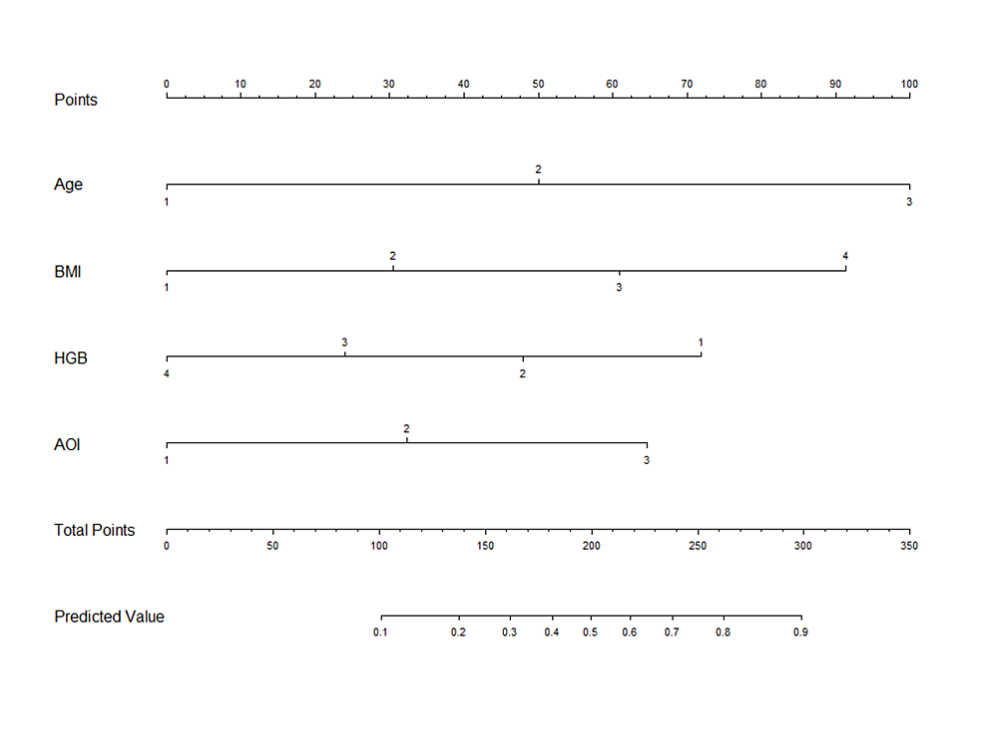
Predictive nomogram model for DVT in pelvic and acetabular fracture patients treated conservatively DVT: deep venous thrombosis, BMI: body mass index, HGB: hemoglobin, AOI: associated organ injury

After the statistical analysis, the following variables were considered for inclusion in the multivariable analysis as candidate predictors: age, BMI, AOI, ASA score, HGB, RBC, and D-dimer. Ultimately, age (OR, 3.04; 95% CI, 1.76-5.26; P<0.001), BMI (OR, 1.97; 95% CI, 1.22-3.18; P=0.006), AOI (OR, 2.05; 95% CI, 1.07-3.95; P=0.031), and HGB (HR, 0.59; 95% CI, 0.39-0.88; P=0.010) were identified as independent predictors of DVT. The nomogram predictive model for DVT was further developed using these four statistically significant screened variables (Figure 1).

Each predictor was assigned a weight coefficient for the score. The total scores for each patient were calculated using the nomogram, which indicated an estimated probability for predicting DVT in pelvic and acetabular fracture patients treated conservatively.

Model performance

**Figure 2:**
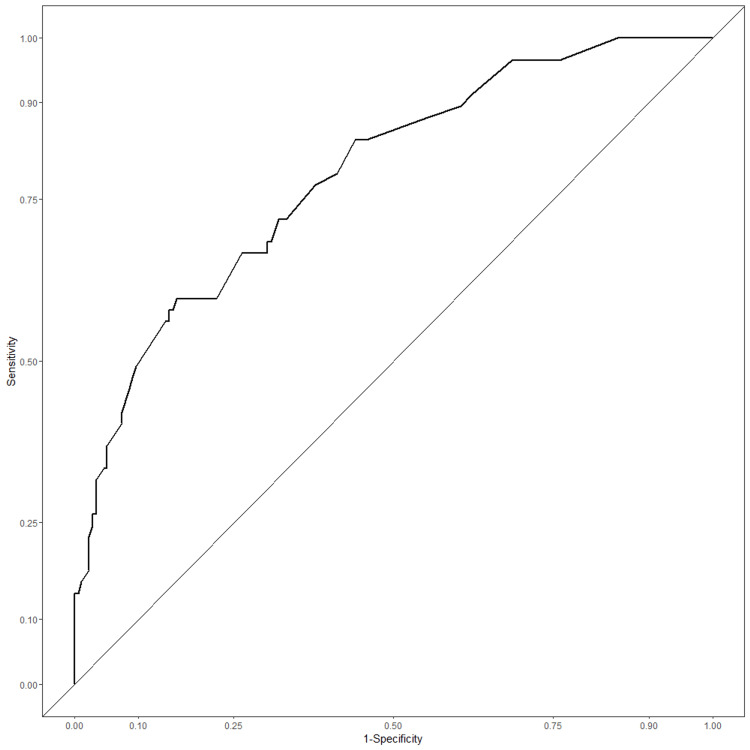
ROC curves for the prediction of DVT in patients with pelvic-acetabular fractures treated conservatively ROC: receiver operating characteristic, DVT: deep venous thrombosis

The discrimination of the predictive model was evaluated by the apparent C-statistic with a value of 0.787, showing good discriminatory ability (Figure 2).

**Figure 3:**
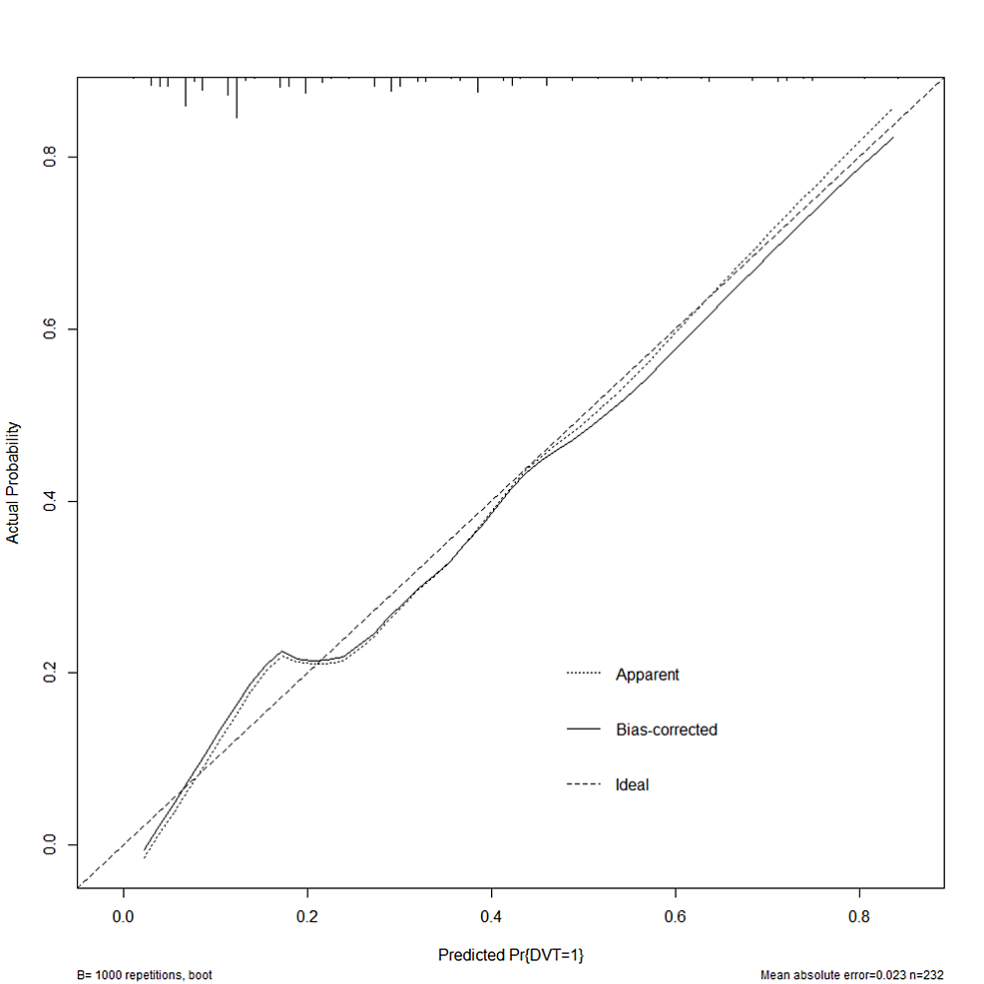
Calibration curves of the nomogram. The solid dashed line represents the performance during internal validation by 1,000 bootstrapping

To eliminate the overfitting performance of the model, 1,000 bootstrap resamplings were performed. The corrected c-index value was 0.753 after internal validation. The calibration plot and Hosmer-Lemeshow test were used to estimate the model's calibration, which indicated a good fit with a P-value of 0.773 (Figure 3).

Clinical practice

**Figure 4:**
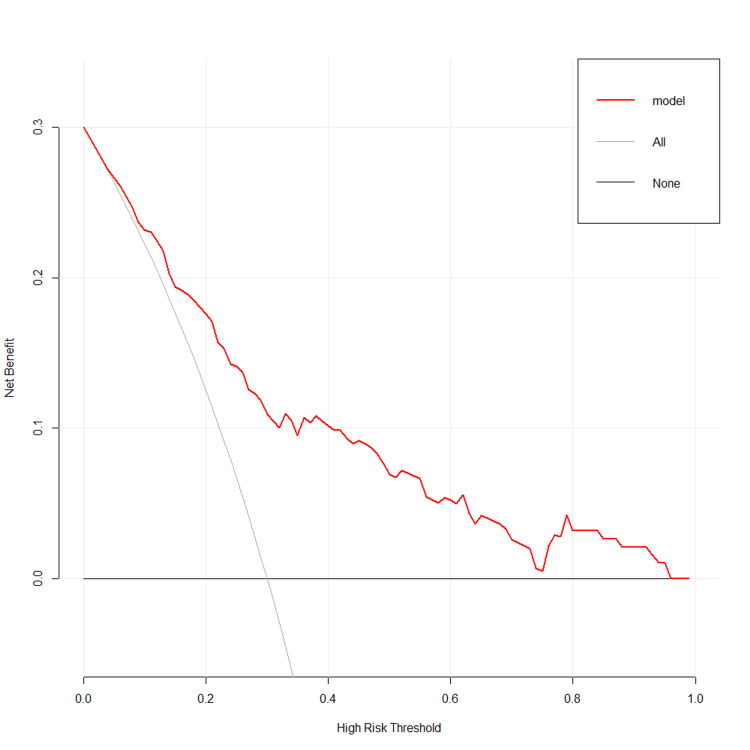
DCA of the nomogram DCA: decision curve analysis

DCA of the nomogram was subsequently performed (Figure 4).

The curve in gray represents the benefits patients got from receiving all precautionary interventions, including taking anticoagulants, using intermittent pneumatic compression devices, or performing regular B-ultrasonic observation. The curve in black represents the benefits gained by receiving no precautionary intervention. According to the predictive model, setting the predicted probability threshold between 0.05 and 0.95 would yield a superior net clinical benefit for the patients.

## Discussion

To the best of our knowledge, this is the first research focusing on risk factors for DVT in patients with pelvic and acetabular fractures who underwent conservative treatment. Additionally, we developed and validated a simple and convenient nomogram model for forecasting DVT occurrences. This innovative model relies on four accessible clinical variables: age, HGB levels, BMI, and AOI.

Age is a well-known factor in various diseases, with mortality and morbidity rates generally higher in older groups compared to younger ones. Previous reports indicate that the annual rate of venous thrombosis increases by approximately 0.5% with age in older patients [7]. An epidemiological study by Stein et al. showed that the DVT diagnosis rate in patients aged 20 to 69 years was about 160 per 100,000, which increased to as high as 655 per 100,000 in patients older than 70 years [8]. Our study reaffirms the significant role of age in increasing thrombotic risks, with older individuals exhibiting higher susceptibility to DVT compared to younger groups (OR, 3.04; 95% CI, 1.76-5.26; P<0.001). These findings underscore the need to focus greater attention on DVT risks in elderly patients.

Another important factor in our study is the role of AOI in the development of DVT. Organ injuries, typically associated with severe trauma, increase the likelihood of a hypercoagulable state. Hypercoagulability, a key component of Virchow's triad of stasis, further elevates DVT risk. The study by Chapman et al. suggested that trauma patients with solid organ injuries transition to a hypercoagulable state as shown on serial thromboelastographic tracings approximately two days post-injury [9]. The coexistence of organ injuries and fractures can synergistically increase the risk of venous thrombosis. Previous research has largely overlooked the impact of AOI [10-14]. In our study, we identified this as a crucial predictor. The risk of DVT roughly doubles with each additional level of AOI. Our findings suggest that therapeutic strategies and post-trauma care should focus more on reducing DVT risk, particularly in patients with organ injuries.

The association between BMI and DVT risk is well documented in numerous studies [15-18], and our research further underscores this relationship. Elevated BMI is not merely an indicator of obesity; it also serves as a surrogate marker for metabolic health and is often linked with a variety of diseases. Our study has shown that patients with pelvic and acetabular fractures treated conservatively who are obese face a significantly higher risk of DVT.

The other critical factor highlighted by our study is HGB levels, which are often overlooked in clinical settings. A retrospective analysis by A. Gu et al. examined the correlation between anemia and complications in patients undergoing total joint replacement [19]. Their findings indicated that anemia was independently associated with various complications, including PE and DVT. However, they did not delve into whether blood type, HGB levels, or RBC transfusions played a significant role in DVT development. A propensity score matching study revealed that patients with femoral and pelvic fractures who received perioperative RBC transfusions were twice as likely to develop DVT as those in the non-transfusion group [20], with the risk increasing in correlation with the volume of transfusion received. Further research is needed to explore the specific indicators and molecular mechanisms by which anemia leads to an increased risk of DVT.

Limitations

The current study has several limitations. Due to its retrospective design, the potential for information or temporal bias is unavoidable. Moreover, being a single-center study, the nomogram model requires further validation in a larger, multicenter clinical study to confirm its generalizability.

## Conclusions

Our research provides a thorough understanding of the factors relevant to DVT in patients with pelvic and acetabular fractures treated conservatively. The nomogram model developed in our study could serve as a practical tool and be applied in clinical practice. It could assist physicians in making decisions and potentially reduce the incidence of DVT.
